# Mining in the Newspapers: Local and Regional Media Representations of Mineral Exploration and Mining in Finland, Germany, and Spain

**DOI:** 10.1007/s42461-021-00453-4

**Published:** 2021-07-06

**Authors:** Jari Lyytimäki, Ludger Benighaus, Javier Gómez, Christina Benighaus, Sari Kauppi, Juha M. Kotilainen, Tuija Mononen, Virginia del Rio

**Affiliations:** 1 Finnish Environment Institute, Helsinki, Finland; 2 Dialogik, Stuttgart, Germany; 3 AT Clave, Seville, Spain; 4 University of Eastern Finland, Joensuu, Finland

**Keywords:** Mineral exploration, Media debate, Mining, Newspapers, Risks, Social license to operate

## Abstract

The understanding of public debates over mineral exploration and mining largely originates from exceptional situations such as mining accidents of conflicts. Less is known about how mining is portrayed and understood under more conventional settings. What storylines dominate the local day-to-day public debate? This article presents results from a comparative case study focusing on newspaper coverage of mineral exploration and mining in three European countries representing different geological and socio-economic contexts. Newspaper articles from the Geyer-Erzgebirge region in Germany, the Andalusia region in Spain, and Northern Finland are studied. The sample looks into the period between September 2018 and February 2020 and shows that regional newspapers report about mining issues relatively intensively even in the absence of major accidents or other media events causing peaks of attention. The tone of the articles is generally neutral to positive towards mining activities, reflecting the specific local settings, historical experiences, and future expectations. Despite the different contexts of the three countries, there were considerable similarities to the topics highlighted, including common themes of mining revival, mining events and social interaction, history of mining, and damages related to mining. Past, present, and future employment opportunities related directly or indirectly to the mining sector are key storylines. Another recurrent underlying theme is the need to balance environment and safety risks and socio-economic prosperity, typically covered through ordinary disputes among the mining sector, public authorities, regional non-governmental organizations, and local initiatives.

## Introduction

The responsibility and sustainability of the mining sector have been publicly discussed under two general narratives. One narrative focuses on the economic importance of the sector and its fundamental role as a provider of key raw materials needed for the basic functions of the economy [1–3]. This narrative has traditionally emphasized the gradual accumulation of wealth and prosperity through the utilization of natural resources by manufacturing and industries. More recently, this narrative has also highlighted the need for rapid socio-technical change to meet the sustainability challenges and the role of the mining sector as the provider of rare earth metals and other critical resources needed to transform societies to become carbon–neutral [4–6]. For example, it has been cautioned that the contribution of wind power and solar photovoltaics to the EU transition to green energy may be limited due to shortages of several critical materials [7]. It is stated that the recycling of materials and substitutes obtained from synthetic or biogenic renewable materials will not be able to entirely replace the extraction and utilization of non-renewable mineral deposits in the foreseeable future.

The other narrative is more critical towards the mining sector. It casts light on harmful environmental and social impacts, accidents, and risks. Heated debates over planned, operating, or closed mines direct criticism towards the whole sector and underline the need for responsible practices during all phases of mining [8, 9]. The narrative includes both actual effects and risks, often making claims about insufficient regulatory frameworks, poor public accountability, a lack of transparent and fair governance, an unfair division of benefits, corruption, and a neglect of the rights of indigenous people and livelihoods of local communities [10–13]. Critical discussions around mining taxes and royalties are also a part of this narrative. In order to address these and other concerns, concepts such as “green mining” or “social license to operate” have been introduced and procedures concretely supporting “sustainable mining” have been developed [14–16]. However, under this narrative, such initiatives can be considered as attempts to legitimize the use of natural resources without properly ensuring democratic processes at the local level and a “right to say no” of the local citizens [17].

Media is a key arena where these narratives are constructed and reproduced. Traditionally, newspapers have played a key role as gatekeepers of public debate and arenas of interplay between news sources, journalists, and audiences. However, relatively few research articles focus on the newspaper coverage of mining [8, 18–22]. More studies use media reporting as one source of data complementing document analysis, interviews, or public surveys [9, 23–27]. While such mixed-material studies are certainly useful, they are unable to paint a larger picture of the media debate and its potential influence. These and other previous studies of mining debates have mainly focused on narrowly defined and isolated case studies [28, 29]. They have provided a rich understanding of the highly variable contexts of mining debates and have provided valuable lessons on possibilities to prevent problems [30–32]. However, such lessons are often closely tied to a certain local context, and our understanding of what lessons may be more widely applicable remains thin. As the mining industry is increasingly international, there is a pronounced need for comparative studies allowing learning from multiple cases.

Previous studies have typically focused on public debates during exceptional circumstances, such as the aftermaths of mining disasters or other short periods of heated controversies [3, 8]. Focusing on such extreme cases is important in order to learn how to avoid such situations, but it is also important to better understand what kind of storylines and topics emerge or dominate the debate during the longer periods of non-heated routine media reporting that sets the baseline of public and policy agendas. Framings and tones of such conventional or everyday reporting influence the long-term development of public opinion and awareness that strengthens or erodes trust towards the mining sector. This, in turn, influences policy decisions and willingness to invest.

Furthermore, previous studies indicate that the public mining debate may not be as polarized as it may be assumed based on the high-profile controversies. Polls and studies aimed to determine the medium citizen position towards mining, within the European Union and in other contexts like Canada or Australia, suggest that mining activities are mostly tolerated if they adjust to local governance [33–35]. Trust is clearly a key factor for mining acceptance, but the role of media coverage deserves further attention.

This research aims to contribute to filling these gaps by focusing on the media debate of mining from a comparative case study and routine reporting perspective by asking:

- How does the regional and local newspaper coverage address mining and mineral exploration activities in three European case regions?
- What storylines dominate the debate, and what are the main topics in each case region?
- Who are the key actors presenting their views?
- What is the tone of the news coverage towards mining and exploration?
- How are different risks and opportunities of mining and exploration brought up?

The next section briefly outlines the cases studied here and describes methods for data collection and analysis, followed by a presentation of the results and discussion. Finally, conclusions summarizing key results are presented.

## Methods and Materials

Our materials originate from the INFACT project (Innovative, Non-Invasive and Fully Acceptable Exploration Technologies) that aimed to develop socially accepted, environmentally friendly, and technologically advanced methods for raw material exploration in the European Union. The project conducted detailed studies in three European mining regions. The selected case regions represent different geological, political, and socio-economic contexts but share a cross-national EU framework. The combination of regional cases was designed to allow an examination and comparison of differences and similarities across different contexts of mining and exploration. The cases are the Geyer-Erzgebirge region in Eastern Germany, the Andalusia region in Southern Spain, and the Sodankylä region in Northern Finland [33]. The analysis presented here focuses on regional level newspaper coverage covering these and adjacent areas. Regional level newspaper coverage was considered as the best basis for the analysis since exploration and mining are place-based activities with considerable regional and local social, economic, and physical effects, as well as high awareness among the local community [36, 37].

In addition to geological differences of the sites, the three regions represent different economies, policy systems, and media systems that provide distinctive contexts for public mining debate. The media and communication systems in Germany and Finland have been grouped under the democratic corporatist media model [38]. In this system, the media is coupled with a strong welfare state and democratic corporatism [39]. Media’s autonomy is highly appreciated. Newspapers are privately owned, and the societal role of regional and local press has been relatively strong. Ownership of the commercial media has been somewhat concentrated and public service broadcast companies have an independent and strong position. The media system of Spain has been labelled as a polarized pluralist model typical for relatively young democracies, countries with strong government intervention in economy, or countries that have elite-oriented press [38]. In this polarized model, journalism is less professional and the links between political actors and journalists are strong. The communication industry is dominated by big multimedia corporate groups, coexisting with fast-growing independent digital newspapers.

Despite the increasing importance of social media, the fragmentation of the media industry, and the decline of readership of most printed newspapers, regional newspapers still play a key role as hubs connecting top-down and bottom-up information flows [9, 40]. Newspapers have been widely read, and the societal role of high-quality newspapers is still relatively strong in Finland and Germany [41, 42]. Spaniards have a low level of trust towards the media, especially in comparison to Finland [43]. Spain is considered to have a satisfactory situation of press freedom ranking (ranked 29 by the World Press Freedom Index 2020 [44]) close to France (34) or the UK (35), but far from Finland (2) or Germany (11).

Newspapers influence the public and policy agenda by presenting or omitting certain topics and by framing the presented topics in a certain way [45, 46]. While these are not necessarily conscious choices made by the media entities, they define the topics for the public discourse. The agenda-setting function of the newspapers means that those issues that are highlighted by the news are likely to be the most influential and societally salient [47]. Framing denotes to the practices of news production that highlight (or omit) certain aspects of a perceived reality to intentionally or unintentionally promote a certain problem definition, causal interpretation, moral judgement, or policy recommendation [48].

The sample studied here covers an 18-month period from September 2018 to February 2020. Common procedures for collecting and analyzing the data were agreed on in order to ensure the best possible comparability among the three cases, but flexibility was needed to enable efficient data collection and analysis, considering the different media systems, differences in mining vocabulary and expressions in three languages, and the availability of news articles via electronic databases. Materials include news stories, editorials and columns by journalists, and opinion pieces by the representatives of the public and the stakeholders.

The German case included media articles on mining, raw material, and exploration, focusing on the regional level of “Erzgebirge/Saxony” in East Germany, an old mining region in transformation. Here, direct access to the online newspaper and supplementary through the news platform “Genios” provided the data. The data originated from the regional newspaper “Freie Presse,” using the German keywords for “mining,” “mineral exploration,” and “raw material.” After screening out irrelevant hits, a total of 214 media articles with full-text access were examined.

The Spanish case focused on the Huelva and Seville provinces of the Autonomous Region of Andalusia in southern Spain, a traditional and active mining region. The newspapers consulted included local level newspaper “Tinto Noticias” and regional newspapers with readership focusing on more populated areas within these provinces. These regional newspapers are “Huelva Informacion” and “Huelva Ya” for the Huelva province, and “Diario de Sevilla” and “ABC de Sevilla” for the Seville province. The data was screened by using the Spanish keywords for “mining” and “mineral exploration.” A total of 177 articles were gathered for the selected timeframe.

In Finland, the regional-level newspaper coverage of Northern Finland, a relatively new mining region, was screened based on the “ePress” database. The newspapers were available as digital replicas of the original printed ones. The regional focus was limited to Finnish Lapland, resulting in hits from the following newspapers: “Kittilälehti,” “Koti-Lappi,” “KotoSalla,” “Kuriiri,” “Lapin Kansa,” “Lappilainen,” “Lounais-Lappi,” “Luoteis-Lappi,” “Pohjolan Sanomat,” “Uusi Rovaniemi.” Most hits originated from the leading regional newspaper “Lapin Kansa” that is read mostly in northern Finland where most of the mines of the country are located. It publishes six issues per week, while most of the other newspapers have a smaller circulation and publish one or two issues per week. Based on the testing of different search strings, the Finnish equivalent for “ore prospecting/exploration” was considered to be the most suitable one. Search strategies focusing more widely on mining were unfeasible because they produced a sample that was too wide and unfocussed. Furthermore, initial screening suggested that the news items mentioning exploration often have mining as their main focus. The Finnish sample included 142 articles.

The sample from all three regions allowed charting of the overall volume and temporal development of the mining debate, as well as identification of the main topics presented. Because of the heterogeneity of the materials, a qualitatively oriented content analysis [49] was employed in order to identify the tone of the news item towards mining, key actors having a voice, and the specific topics highlighted. The categorization of the tone of the articles was based on simple categories of “positive,” “neutral,” or “negative”" in order to secure reliable interpretations and comparability between cases and with earlier research [9, 37]. The key actors that were present in the news were coded from the texts and aggregated to a higher level in order to allow country comparisons. Identification of the key topics involved subjective assessment focusing not only on the manifest content but also latent content of the news items and visual materials, when available [18]. Interpretations were based on iterative rounds of reading by researchers and common discussions aimed to produce a shared understanding of the correspondence of topics identified across different countries.

## Results

### Overall Characteristics and Volume of the Coverage

**Fig. 1:**
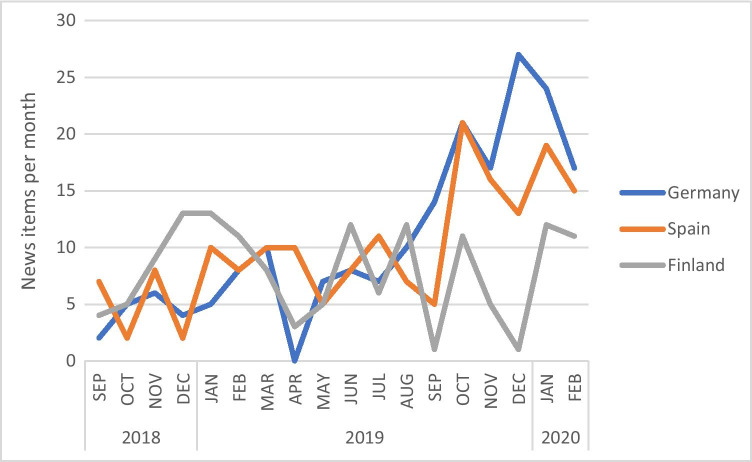
Overall development of regional-level coverage of mining by newspapers in Finland, Germany, and Spain

In all three countries, mining issues were addressed by the newspapers on a regular basis without significant breaks (Fig. 1). The volume of reporting tended to increase over time, but due to the relatively short study period, trends should be examined with care. It remains open whether the trend will continue, peak, or drop for any reason or incident.

In Germany, approximately 12 articles of the newspaper “Freie Presse” addressed mining and mining-related topics per month. The number varied from 0 in March 2019, to up to 27 articles in November, and 24 in December 2019. The peak was partly influenced by the Christmas time and the festive events around mining, which take place at the end of each year.

In Spain, on average, 2 news items were published per newspaper and month (10 articles per month). The trend during the 18-month period was influenced by peaks covering certain relevant events, accidents, and lawsuits, like the III International Mining and Minerals Hall in Seville (October 2019).

Also, the results from the Finnish case — based on a narrower focus on exploration — suggest that the regional level newspapers cover mining and mineral exploration regularly. The regional newspaper “Lapin Kansa” published, on average, over four (4.3) news items per month mentioning or focusing on mineral exploration. The coverage was characterized by fluctuations related mainly to the activity of exploration and mining firms and related administrative processes and societal debate over mining issues such as the national mining law.

### Tone of the Debate

**Fig. 2:**
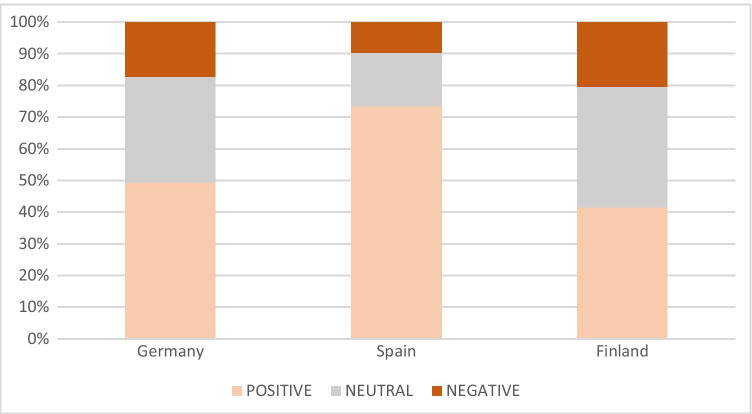
Tone of the coverage of mining by regional newspapers in Finland, Germany, and Spain, Sep. 2018–Feb. 2020

Most of the news items were characterized by a positive or neutral tone towards mining and exploration (Fig. 2). Nearly half of the 214 German articles gave positive messages. Roughly one-third of the tone could be characterized as “neutral,” while 17% were clearly on the negative side. Likewise, in Spain, three out of four news were positive, while only about 10% had a negative tone towards mining. The strong historical, social, and economic links between the mining sector and the local stakeholders were a key explanation for the rather benevolent attitude towards mining in regional media. As examples, in Spain, the legal aspects of a partially failed environmental authorization of a mine were covered by many articles, some quite illustrative: “[Locals] claim the environmental authorization has been paralyzed for 400 days” (TintoNoticias, 21 January 2020), “Miners and mayors threaten to protest if the authorization is not solved soon” (TintoNoticias, 9 January 2020), “[Union] joins defense of mining activity in [the region]” (TintoNoticias, 2 July 2019) or “[Political party] urges for the granting of the authorization of the mine” (HuelvaYa, 20 January 2020). There were many positive articles covering the International Mining Hall celebrated in the region as well, e.g., “[Regional capital] mining global showcase” (Diario de Sevilla, 15 October 2019).

In Germany, a number of articles focused on the new era of mining, which is often positively framed. Headlines like “First steps for the return of mining” (Freie Presse, 18 September 2018), “Saxony will remain a mining country” (Freie Presse, 17 June 2019), and “Bergeschrey will shape Luchsbach valley again for decades” (Freie Presse, 10 April 2019) showed a neutral to positive tone in media. However, a mixed tone with both positive and negative (concerned) arguments and news was linked to the new mine in Poehla, as these headlines indicate: “The ore mine in Pöhla will mine tungsten and fluorspar from 2021” Freie Presse, 3 July 2019), “Ore mine/city insists on their interests and those of the citizens” (Freie Presse, 13 April 2019), and “Mining plans in Poehla move people” (Freie Presse, 28 February 2020).

In Finland, the share of news items with a neutral tone was high. The results suggest that the share of news items with a positive tone towards exploration and mining decreased in 2019 if compared with 2018. However, the two first months of 2020 showed a clear dominance of positive tone (52% of the coverage) and a sharp decrease of coverage with a negative tone (to 9%). This was mostly explained by the lack of critical opinion pieces and several editorials supporting the mining industry by the regional newspaper “Lapin Kansa.” Overall, the editorials written by the senior journalists were dominated by a positive stance towards mining, for example, a piece making a direct plea highlighting the expected economic and employment gains: “Let’s not expel the mines,” (Lapin Kansa, 7 June 2019).

### Key Actors of the Debate

**Fig. 3:**
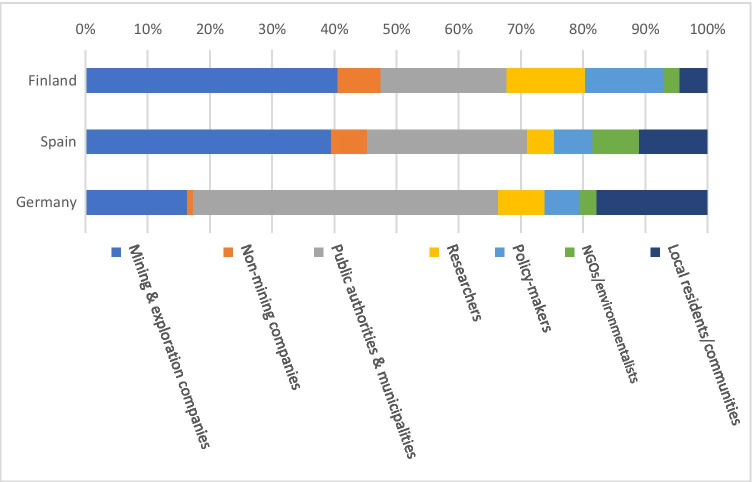
Key actors present in the coverage of mining by regional newspapers in Finland, Germany, and Spain, Sep. 2018–Feb. 2020

Key actors having a voice in the newspaper differed somewhat between the countries (Fig. 3). In the German case, public authorities and municipalities dominate the sample as key actors. This result suggests that mining (including all phases of mining, historical, recent, and future prospects) is strongly governed by the local level and by authorities (e.g., institutions in charge of mining, such as Oberbergamt Freiberg). Surprisingly, only a few mining companies were cited in the news, the one starting a new mine at Poehla dominating the scene. The tone towards the mining company varied over time but had clear downward tendencies due to results of a public hearing and critical requests from the local community. Furthermore, the location of the shaft which will be used later for the underground mine, and the effects on the landscape, traffic, and environment raised concerns.

In the Spanish case, the mining companies were the most salient stakeholders, followed by public authorities. Also, the local communities were quite frequently cited, mostly because of the local and regional scope of the newspapers. The visibility of environmentalists, mining institutions, unions, and academy was much lower, but their views were contrasted against the major actors. Regional media recurrently featured actors related to mining and mining institutions, events, investments, policies, and budgets. On the one hand, the local community and stakeholders demanded to be informed frequently about mining. Mining is not just an economic activity in the region, but part of the identity of its inhabitants. On the other hand, this could be explained by the mining companies’ desire to influence the public agenda to maintain a positive attitude towards mining.

In Finland, the mining and exploration companies were the most prominent group of actors, followed by regional-level authorities, and representatives of municipal administration and policymakers. The public visibility of environmental non-governmental organizations (NGO) was weak. In many cases, the exploration and mining firms were able to strongly influence the agenda-setting by being the only party interviewed by journalists. Short news was often based on the press releases and other communication material provided by the exploration companies, while longer news items included interviews of the representatives of the companies and occasional comments from local or national policymakers, authorities, and researchers. The tone of short pieces was typically neutral, while longer pieces presenting commentaries from representatives of exploration or mining companies often had a positive tone. Interestingly, even though economic importance and the potentials of mining were often referred, the actual assessments of the local, regional, or national economic impacts of mining were almost completely missing from the debate.

Local community was cited and present in all three regions, but had a secondary role. Local concerns were addressed by media, but usually, they were introduced by other actors, mainly public authorities. Other stakeholders present in media debate are academy and research institutions, together with environmental NGOs, either supporting certain argumentations or pushing for their own agenda. Unions and political parties were only relevant in Spain, suggesting a more politicized discussion than in the other countries.

### Key Topics and Storylines

Several common topics were identified between the countries, while some key topics of the debate reflected specific country contexts (Table 1). In Germany, due to the long and rich history of mining in the region of Erzgebirge and the highly active and successful use of tourism potential, the most common topics included historical mining in general, specific events for mining (often associated with the history of mining), and UNESCO world heritage, which means that parts of Erzgebirge belong to the world list of most important heritage sites. Damages from old mining was another topic high on the agenda and indicated the long and costly restoration of mining projects and remains dating from the middle ages up to the recent past. The popularity of the topics new mining in the ore mountains (11% of the articles) and exploration (6%) indicate, on the one hand, hopes for a new era of mining, and an increasing importance in different sectors, and, on the other hand, tensions between society and key actors with the mining area. The topic of new mining in the ore mountains addressed general issues, such as the potential for new mines or the chances of geothermal power and the long-lasting reputation of a traditional mining region. One specific project was addressed frequently (9%). The development of a new underground mine in the municipality of Poehla was described through articles informing the citizens about the exploration phase, about public events, and also reporting about complaints, fears, and hopes for the future and what a mine means for the local community.

**Table 1:** Key topics of the coverage and examples of key issues of mining debate by regional newspapers in Finland, Germany, and Spain, Sep. 2018–Feb. 2020

	Finland, Lapland	Germany, Geyer-Erzgebirge	Spain, Western Andalusia
Topics presented in all three countries			
New mines	Speculations over several new mines or reopenings	New mining in the Ore Mountains	Aznalcollar mining reopening
Events and social interaction	Interaction between the local stakeholders and mining sector	Events for mining	International Mining and Minerals Hall in Seville
History of mining	Mining heritage especially related to small-scale exploration	Historical mining in general, UNESCO world heritage	Mining heritage
Accidents and damages	Damages as a latent theme	Damages from old mining	Cobre Las Cruces landslide
Technology	New technologies, batteries industry, energy transition, critical minerals	Research, innovation	Innovative mining-related activities
Environment and permits	Future ecological risks, environmental permits of exploration	Nature, environment	Atalaya’s RioTinto environmental permit
Women’s career	Women in mining-related professions	Women in mining	Women in mining
Topics highlighted in two countries			
Mineral exploration	Technologies and processes of mineral exploration	Exploring raw materials	
Skills and education	Lack of skillful workforce, education needs	Education	
Legal issues	Renewal need of the Mining Act		Lawsuits, corruption
Economic importance	National level economic importance of mining sector		Mining company’s economic growth and investments
Regional development	Potential for regional level employment and well-being		Mining and long-term territorial development
Other topics relevant only in a single country			
Fairness	Fairness of resource use, domestic versus foreign	Coal production	Historic mining coal

The main topics in the Spanish newspaper coverage were lawsuits, mentioned in 46% of the articles, followed by mining revival (32%), and the safety of mines (24%). Safety issues were increasingly mentioned towards the end of the study period. The media treatment of the other topics remained at a constant level. Mining dependency awareness and mining decline were secondary topics. Somewhat surprisingly, past mining accidents were not a major topic despite the legacy of the infamous Aznalcollar disaster. This was the worst environmental catastrophe in Spain in recent times, caused by a dam accident that released mining wastes into the Guadiamar River, one of the main water sources of the wetlands of Doñana National Park [50–52]. Instead, more attention was given to current issues, such as lawsuits directed at Rio Tinto mining company, Atalaya mining, as a result of an incomplete environmental permit process in 2019. Most of the attention was directed at metallic mining, while exploration received less attention.

Because of the selected search strategy, the mineral exploration was the most prominent (55% of the articles) topic in the Finnish sample. However, a substantial share of the news items (31%) focused on mining and only mentioned exploration in passing. Both types of news generally emphasized the importance of the mining industry for the economy and employment and often discussed the interactions between the mining sector and the rest of the society. The news items that had exploration as their main topic most often informed about permit decisions for plans to study certain areas. These items were typically short news based on announcements by the exploration firms. News about concrete progress of the exploration process was less prominent. Safety issues related to the mining industry were not present in the sample. Likewise, coverage specifically focusing on past mining accidents was missing from the sample, despite the importance of the recent problems and accidents in the infamous Talvivaara mine in Eastern Finland [8, 27]. Occasional news items related to legal processes typically described complaints against permits for exploration. Discussions over the need to renew the Finnish mining act brought up related fundamental questions of ownership of mineral resources and fairness of the distribution of socio-economic benefits.

More detailed observations revealed some interesting insights. In Finland, several front-page news and long reportages with a positive tone towards exploration were published. For example, one two-page reportage titled “The smell of gold wafts from the deeps of the fell” introduced two persons from a Canadian exploration firm. The comments by these interviewees built highly positive framings of exploration with strong connotations of gold mining as a desirable and beneficial activity. The reportage reproduced the myth of a gold rush and evoked emotive responses often attached to the utopian views of northern resources [53] by depicting Finnish Lapland as a place of unimaginable richness: “Here you can accidentally trip and fall because of a piece of stone containing gold” (Lapin Kansa, 9 August 2019). In Spain, the most positive news items were linked to the celebration of the International Mining and Minerals Hall in Seville, as well as the reports on the mining company’s economic growth and investments. In Erzgebirge, Germany, the mineral exploration at Poehla and the operational activities to open a new mine brought about many critical viewpoints from the media. This demonstrates how easy and quickly a public debate can turn negative.

In all three countries, environmental risks were often mentioned in passing, or they were a latent theme of the discussion. The focus was on potential future risks while only a few items specifically focused on current or past environmental effects of mining. The actual environmental effects of exploration received relatively little attention. In addition to environmental risks, the environmental benefits of the mining industry were discussed. In Finland, one sub-theme of the topic of mining revival was the importance of minerals for the development of an emerging battery industry cluster and, more widely, for the transition of the whole energy system. Women’s career was a topic present in varying degree in all countries, but, generally, not dominating the debate. Actors being cited in the news were typically male and women were presented as external actors joining the mining sector, or being underrepresented. For example, in Spanish newspapers, the focus was on training programs for women and emerging women self-empowering initiatives within the mining sector (“The association Women in Mining and Industry Spain comes into existence in Huelva,” Huelva Ya, 11 February 2020).

## Discussion

Media representation is one key part of the complex societal dynamics affecting the public image and social acceptance of the mining sector. Media, policy, and public agendas are intertwined in different ways in different societies, and it is, therefore, challenging to draw general lessons. However, our cases indicate that some topics are reported regularly, while others are more likely to be addressed irregularly or only occasionally. Our cases suggest that the mining news regularly feature issues related to employment, investments, and social events.

The results also suggest that ordinary reporting of mining issues often characterized by neutral or positive tone differs from reporting focusing on specific extraordinary events characterized by negative tone. High-profile events such as lawsuits or mining accidents are characterized by rapidly fluctuating coverage and a potentially long shadow of suspicion and mistrust as indicated by the Talvivaara and Aznalcollar cases [8, 52]. This highlights the need to take the specific history of risks into account when communicating about present activities [54]. Past experiences of mining are reproduced by the news and influence current perceptions partly regardless of the current practices of exploration and mining companies [11, 55]. The cautionary tales from history easily become overemphasized because the public generally lacks knowledge of the latest technologies such as non-invasive exploration technologies [34]. Also, the criticism directed towards specific cases and accidents is easily perceived as criticism towards the entire mining sector.

Mining revival was a key general theme in all the countries. Newspapers covered it in different ways, from broad reportages to focused storylines describing exploration activities, plans for new mines, private investments, innovative mining-related technologies, and employment or education opportunities. The Finnish newspapers reported on exploration in various locations and presented speculations over several potential new mines or the reopening of old mines. This was different from German and Spanish coverage of new mines focusing more on the future potentials of a limited number of certain individual mines. Mining revival raised concerns regarding social issues, economic implications, and safety and environmental risks. They were addressed in all the three case regions from a double perspective. First, there was a short-term point of view highlighting environmental damages and accidents, as well as the current issues related to societal fairness and inequalities perceived (gender gap, legal issues, and distribution of economic gains). Second, there was a long-term point of view on the integration of mining activities into a successful model for regional development. The future scenarios discussed by the regional newspapers were often contrasted with specific historical mining experiences and past accidents. Therefore, future outlooks become deeply linked with the region’s past, heritage, and identity, as well as wider considerations of world markets of raw materials [9, 11]. These include the EU’s concerns of critical minerals and uncertainty of the markets in Asian ore production. The COVID-19 crisis has only deepened the mistrust in Asian markets [56], serving as an example of rapid changes in mining debates.

Overall, the short-term concerns shared more common elements with all three case regions, while the representations of long-term concerns were more versatile. The long-term future outlooks in Finland focused mostly on the knowledge-intensive economy and transition of the energy sector, stressing the importance of critical minerals for information and communication technologies and the prospects of the batteries industry. In Germany, the future outlooks were built based on the long history of mining and its cultural implications, stressing the importance of tourism and the service sector. In Spain, the debate revolved around the mid-term continuity of the metallic mining sector and investment possibilities guiding the region towards a more sustainable and responsible economic model with high employment and resilience to future crisis.

Regarding environmental issues, in Spain, the environmental permit of one single mine (Aznalcollar) was the most referred topic in all newspapers. Environmental concerns were raised for other main storylines as well, linking the reopening of Aznalcollar mine and the accident in Cobre Las Cruces with potential water pollution. While not such a big topic in Geyer Erzgebirge Germany, environmental concerns were raised relating to the new mine in Poehla and its location close to a protected area and nature reserve. In Finland, environmental concerns were mainly brought up by occasional and often indirect references to the notorious Talvivaara mine and by speculations about potential environmental risks of future mining activities. However, contrary to some expectations [9, 46], the regional-level debate was not dominated by a long-lasting confrontation between the mining industry and its adversaries spearheaded by environmental NGOs.

Lawsuits were not frequently covered in Finland or Germany, but were a hot topic in Spain. This was largely coincidental and due to specific legal processes during the study period. In Finland, the debate over a potential need to renew mining legislation was a prominent theme, especially related to the responsibilities of mining firms and the distribution of economic profits and socio-ecological risks. In Germany, this debate was dominated by the transformation of the historical coal mining sector, which is relevant for the region, and the phase-out of mining coal nationwide in the long-term [19, 57, 58].

Despite the negative and neutral news items addressing on-going lawsuits against mining companies, the tone of news items in Spain was most often positive, reflecting the high level of social acceptance and regional support for mining activities. In Andalusia, there is a high interest from public authorities and mining companies on promoting a positive image for the mining sector that leads to the publicizing of investments and funding of events with a strong social component [59]. For Erzgebirge Germany, the tone of the articles was also neutral or slightly positive. This was mainly explained by the focus of the majority of articles on less controversial issues of historical mining heritage and its use for tourism, as well as events attached to recreation activities.

The tone used by the media was more positive towards exploration than towards the entire mining sector. Mineral exploration was mostly represented as an innovative mining-related activity. Exploration was framed as an activity ultimately benefitting the mining companies, but also as an effort conducted by public authorities and academy that may have a beneficial impact on future regional development. In Spain, less than 20% of the mining news during the study period covered exploration. It is a secondary topic in the Iberian Pyritic Belt, where the active mining operations dominate the media debate. Future-oriented coverage focusing on exploration and prospects of new mining or revitalization of old mining areas was more pronounced in Finland and in Germany. In Finland, the news items focusing on exploration had a particularly positive tone, often related to the expectations of economic and employment benefits and the prospects of new energy technologies related to Finland’s aim to be a zero-carbon society in 2035.

Even though the positive views dominated the debate, a polarization between views emphasizing the opportunities for economic and employment benefits of mining and views emphasizing potential negative impacts on nature and local livelihoods was present. Such a polarization can be deepened in the absence of integrating concepts [60]. Environmental risks of mining, potential threats to tourism, and, increasingly, issues related to fairness, liability, and ownership of mineral resources were concerns that were raised. In Finland, editorials written by the newspaper staff were clearly in favor of mining and exploration, while critique was presented mainly in opinion pieces. Journalistic news generally had a neutral or positive tone towards exploration and mining.

Our results support earlier studies showing that a major part of media’s reporting, possibly as high as one-third, is based heavily on press releases produced by various societal actors, including companies [61]. This could mean that at the local and regional levels, exploration companies are able to emphasize the positive economic expectations, while downplaying economic risks or realities, which serves the companies’ interests and needs to portray the project in an attractive manner to the potential investors. This can build unrealistic expectations for the local communities and skew the local deliberation on whether they should accept or reject such projects in their region. Our results suggest that this risk exists both in contexts of the democratic corporatist media model of Finland and Germany and polarized pluralist media model of Spain.

The relatively weak voice of local communities was a surprising result, given the regional and local focus of the newspapers studied here. Also, academics could play a more active role in regional media outlets to increase public engagement on mining issues and related challenges, such as energy transitions and climate change [62]. Especially in Finland, the rather low visibility of environmental NGOs was surprising, given the high visibility of social movements such as Stop Talvivaara and several smaller local movements elsewhere and active opposition towards exploration in the case study area [11]. The reasons leading to actors being vocal or silent in the news clearly requires further investigation. The relatively weak visibility of NGOs may also be because these organizations increasingly focus on influencing through other channels such as campaigning and networking through social media [55, 63]. While the future role of regional newspapers remains unclear, it appears that there is room for a more integrative role bringing different voices together as newspapers are increasingly read through digital platforms.

One caveat in our study is that debate in certain case regions and during a certain time period allows only uncertain assumptions about the debate elsewhere and at other times. Therefore, further comparative analysis and follow-up studies are needed in order to better anticipate the emerging concerns and changes in media framings and public attitudes.

## Conclusions

Whether an issue is highlighted or omitted by the media reporting obviously influences the awareness by stakeholders and general public. In addition, the tones and framings of news coverage have implications on investment decisions and policy priorities. The results presented here suggest that the tone of routine reporting by local and regional newspapers is more positive towards the mining sector than the tone during exceptional situations that typically describe accidents, risks, or controversies. The positive tone of regional-level newspaper coverage reflects not only different mining histories of specific areas, but also different socially constructed perceptions and expectations.

There are two competing but intertwined general storylines that emphasize positive economic and employment opportunities and negative environmental and social implications of mining. These storylines are influenced by stakeholders with pro-mining or anti-mining agendas. The negative storyline is primarily pushed by associations and locals concerned with environmental issues and risks, such as water usage, waste management, and sustainable future alternatives. The negative storyline easily becomes strengthened by isolated cases of accidents and misconducts serving as cautionary examples, affecting the perceptions of the entire mining sector far beyond specific cases.

The positive storyline covers the benefits of the mining sector, including activities of the mining companies at a regional level, the funding of social activities and events. These are related to prospects of economic growth, new investments and employment opportunities, and public support of mining from different local stakeholders. This positive attitude of the media towards mining is partially dependent on the influence of mining sector on local economy, and it may rapidly dwindle if the economic importance of the sector decreases. On a longer-term, the positive storyline may remain persistent because of high and even increasing societal demand for many products of the mining industry. However, if the expected regional benefits are not achieved or decline, for example due to automatization or the use of non-local workforce, while the “environmental costs” continuously make headlines, it is possible that the rationale for accepting mining activities is questioned. This highlights that unless practices and business models that genuinely meet responsibility and sustainability criteria are convincingly implemented, the currently prevailing positive storyline will be increasingly challenged.
